# Gut Symbiont *Bacteroides fragilis* Secretes a Eukaryotic-Like Ubiquitin Protein That Mediates Intraspecies Antagonism

**DOI:** 10.1128/mBio.01902-17

**Published:** 2017-11-28

**Authors:** Maria Chatzidaki-Livanis, Michael J. Coyne, Kevin G. Roelofs, Rahul R. Gentyala, Jarreth M. Caldwell, Laurie E. Comstock

**Affiliations:** Division of Infectious Diseases, Brigham and Women’s Hospital, Harvard Medical School, Boston, Massachusetts, USA; Harvard Medical School

**Keywords:** *Bacteroides*, antagonism, microbiota, ubiquitin

## Abstract

Human gut *Bacteroides* species produce different types of toxins that antagonize closely related members of the gut microbiota. Some are toxic effectors delivered by type VI secretion systems, and others are non-contact-dependent secreted antimicrobial proteins. Many strains of *Bacteroides fragilis* secrete antimicrobial molecules, but only one of these toxins has been described to date (*Bacteroidales* secreted antimicrobial protein 1 [BSAP-1]). In this study, we describe a novel secreted protein produced by *B. fragilis* strain 638R that mediated intraspecies antagonism. Using transposon mutagenesis and deletion mutation, we identified a gene encoding a eukaryotic-like ubiquitin protein (BfUbb) necessary for toxin activity against a subset of *B. fragilis* strains. The addition of *ubb* into a heterologous background strain conferred toxic activity on that strain. We found this gene to be one of the most highly expressed in the *B. fragilis* genome. The mature protein is 84% similar to human ubiquitin but has an N-terminal signal peptidase I (SpI) signal sequence and is secreted extracellularly. We found that the mature 76-amino-acid synthetic protein has very potent activity, confirming that BfUbb mediates the activity. Analyses of human gut metagenomic data sets revealed that *ubb* is present in 12% of the metagenomes that have evidence of *B. fragilis*. As 638R produces both BSAP-1 and BfUbb, we performed a comprehensive analysis of the toxin activity of BSAP-1 and BfUbb against a set of 40 *B. fragilis* strains, revealing that 75% of *B. fragilis* strains are targeted by one or the other of these two secreted proteins of strain 638R.

## IMPORTANCE

We are just beginning to understand some of the important interactions that occur between microbes of the human gut microbiota that dictate the composition and abundance of its constituent members. The ability of one member to produce molecules that directly kill a coresident member has been shown among minor gut species and is just starting to be studied in the abundant *Bacteroides* species. Here, we show that some strains of *Bacteroides fragilis* have acquired a gene encoding a secreted eukaryotic-like ubiquitin protein with potent inhibitory activity against other *B. fragilis* stains. This is the first bacterially encoded ubiquitin-like molecule shown to function like a bacterial toxin. This molecule is an example of a gut symbiont acquiring and adapting a eukaryotic molecule likely to increase its competitiveness in the mammalian gut. Understanding antagonistic factors produced by abundant gut symbionts is an important prerequisite to properly engineer strains to colonize the gut for health benefits.

## INTRODUCTION

The gut microbiota of healthy humans is comprised of many different microbes, with members of the order *Bacteroidales* being the most abundant Gram-negative bacteria. Numerous *Bacteroidales* species colonize the human gut simultaneously at high density, and colonization with more than one strain of the same *Bacteroidales* species is common (1, 2). Some factors that may account for the ability of so many closely related species and strains to colonize the same ecosystem include their ability to utilize different nutrients (3) or to prioritize the utilization of different nutrients (4, 5), their occupation of different spatial niches (6, 7), and their ability to cooperate in the utilization of dietary polysaccharides (8, 9). *Bacteroidales* species also physically contact each other in the human gut and have been shown to exchange more than 100 kb of DNA between strains with the transfer of a single conjugative element (10, 11).

Despite characteristics of the *Bacteroidales* that permit or promote cocolonization, these bacteria have also evolved mechanisms to antagonize each other. Antagonism or interference competition is likely an important factor dictating the composition of diverse microbial communities. *Bacteroidales* have been shown to elicit two different types of antagonistic systems: contact-dependent type VI secretion systems (T6SSs) (12–15) and secreted antimicrobial protein toxins (16, 17). Most *Bacteroides fragilis* strains have genetic loci encoding T6SSs (12), and some of these systems have been shown to antagonize nearly all gut *Bacteroidales* species tested (13). As T6SSs are contact dependent, this antagonism may largely occur when nutritional niches overlap and/or when dietary nutrients are limiting and *Bacteroidales* species are forced to utilize host mucins, one of the preferred carbon sources of *B. fragilis* (5).

In contrast to the *B. fragilis* T6SSs, the two identified *Bacteroidales* secreted (non-contact-dependent) antimicrobial proteins (BSAPs) each targets a subset of strains of the same species (16, 17). Both described BSAPs contain membrane attack/perforin (MACPF) domains found in immune molecules, such as complement components and perforin, that lyse bacteria or virally infected cells by pore formation. BSAP-1 and BSAP-2 are the only bacterially produced MACPF proteins shown to kill other bacteria. BSAP-1 is produced by a subset of *B. fragilis* strains and mediates its toxicity through pore formation following recognition of a specific outer membrane β-barrel protein on sensitive (non-BSAP-1-producing) *B. fragilis* strains. BSAP-2 is produced by a subset of *Bacteroides uniformis* strains and recognizes the lipopolysaccharide (LPS; core polysaccharide or short O antigen [O-ag]) of sensitive (non-BSAP-2-producing) *B. uniformis* strains. The genes encoding both BSAP-1 and BSAP-2 were acquired with adjacent genes encoding orthologs of their receptors, replacing the receptor and rendering the strain resistant to the newly acquired toxin (17).

In studying secreted antimicrobial molecules produced by *B. fragilis*, we found that several strains, such as 638R, inhibited the growth of many *B. fragilis* strains, whereas other strains had no secreted antimicrobial activity against the panel of strains analyzed (16). We also showed that a mutant in which the BSAP-1-encoding gene of *B. fragilis* 638R is deleted retains the ability to inhibit the growth of a subset of *B. fragilis* strains (16). The present study was designed to identify and characterize the additional secreted antimicrobial molecule of strain 638R. Here, we describe a novel eukaryotic-like ubiquitin molecule that mediates potent antimicrobial activity against *B. fragilis* strains.

## RESULTS

### Spectrum of intraspecies antagonism by secreted molecules of *B. fragilis* 638R.

**FIG 1:**
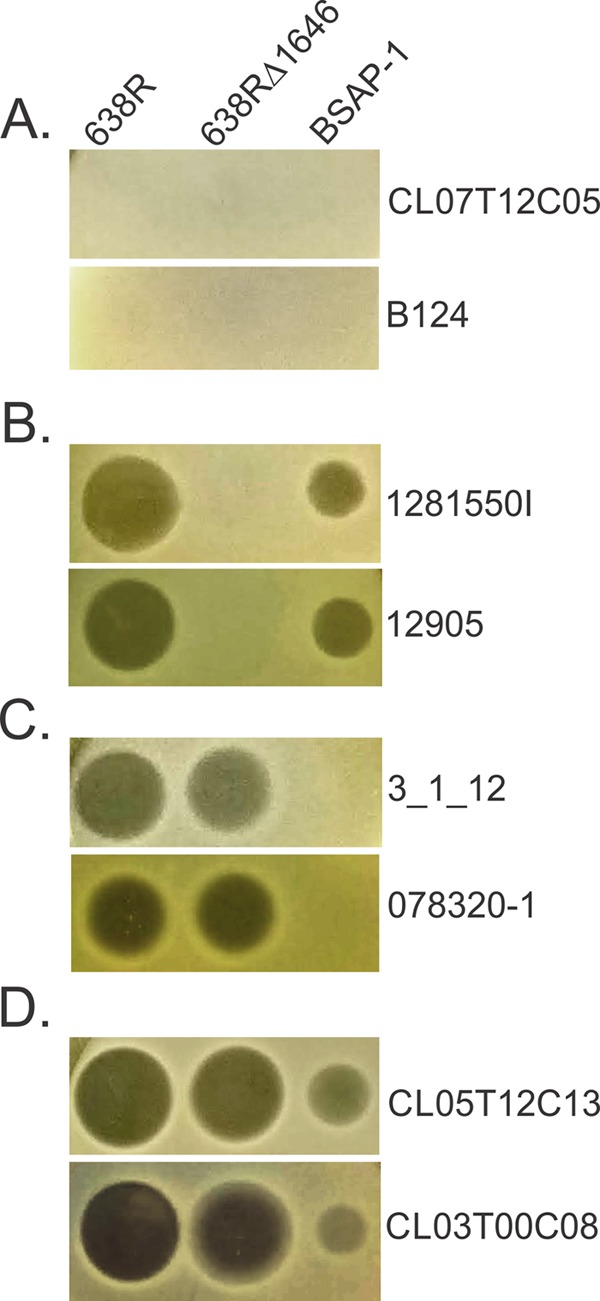
Agar spot assays of eight *B. fragilis* strains in overlays, showing zones of growth inhibition (dark spots) by secreted molecule(s) from wild-type strain 638R and 638RΔ1646 (BSAP-1 deletion mutant) and by purified, His-tagged BSAP-1. Results for two strains each, named to the right of the panels, are shown as examples of the four different patterns of sensitivity/resistance.

We performed a comprehensive analysis using 40 *B. fragilis* strains from our collection to determine their sensitivity to BSAP-1 or other antimicrobial molecule(s) secreted by *B. fragilis* 638R. Partial or complete genome sequences were available for 11 of these *B. fragilis* strains, and we sequenced, assembled, and annotated the genomes of an additional six strains (see Table S1 in the supplemental material). Using wild-type 638R, the 638RΔ1646 (BSAP-1 gene deletion) mutant, and active purified His–BSAP-1, we determined the sensitivity profiles of these 40 strains (Table S1). As shown by the results in Fig. 1, there were four different patterns of sensitivity/resistance. Only seven of these strains were not susceptible to secreted molecules of 638R (Fig. 1A). Ten strains were sensitive to BSAP-1 only, as His–BSAP-1 produced a zone of inhibition and no inhibitory activity remained in the 638RΔ1646 mutant (Fig. 1B). Four strains were not antagonized by BSAP-1 but were inhibited by a different molecule(s) secreted by 638R (Fig. 1C), and 19 strains were antagonized by both BSAP-1 and an additional molecule(s) secreted by this strain (Fig. 1D).

10.1128/mBio.01902-17.1 TABLE S1  Ability of *B. fragilis* 638R, 638RΔ1646 mutant, and proteins to inhibit the growth of *B. fragilis* strains. Download TABLE S1, DOCX file, 0.01 MB. Copyright © 2017 Chatzidaki-Livanis et al. 2017 Chatzidaki-Livanis et al. This content is distributed under the terms of the Creative Commons Attribution 4.0 International license.

### Identification of a gene necessary for inhibitory activity in 638RΔ1646.

**FIG 2:**
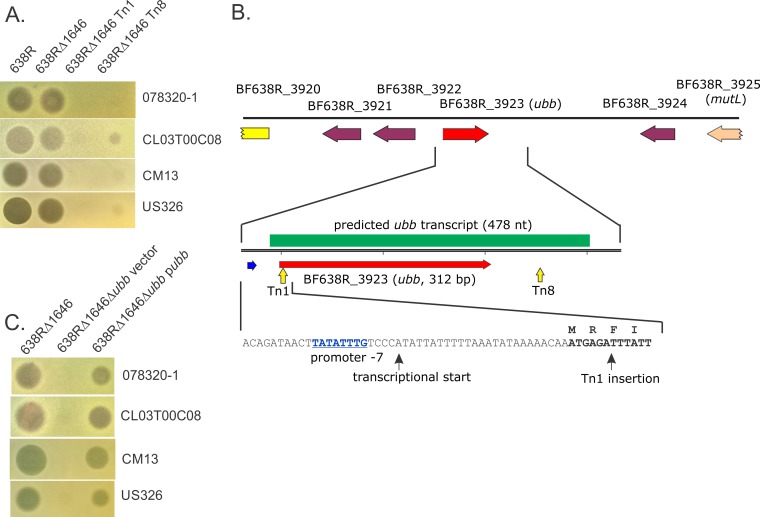
Identification of a gene necessary for antimicrobial activity of 638RΔ1646. (A) Agar spot assays showing results for two transposon mutants of 638RΔ1646 that lost inhibitory activity against four *B. fragilis* strains. (B) (Top) ORF map of the genetic region where the transposons insertions into the 638R genome resulted in loss of activity. (Bottom) Extent of the BF638R_3923 (*ubb*) transcript as predicted from analyses of RNA-Seq data. A perfect match with the −7 site of the promoter sequence recognized by the *Bacteroides* sigma 70 factor is shown (blue letters). (C) Agar spot assays showing the loss of secreted inhibitory activity in a *ubb* deletion mutant and the resulting phenotypes when the gene is added to the mutant in *trans* (p*ubb*), as well as results for the vector control.

To identify the molecule or molecules responsible for the second antimicrobial activity, we performed transposon mutagenesis of the 638RΔ1646 strain and screened for loss of inhibition of the sensitive *B. fragilis* strain CL03T00C08. Two transposon mutants of 638RΔ1646 were identified that were severely attenuated in their ability to inhibit the growth of this strain (Fig. 2A). In both of these mutants, the transposon inserted within the transcribed region of an unusual bacterial gene previously recognized as encoding a very close ortholog of eukaryotic ubiquitin, termed *B. fragilis* Ubb (BfUbb) (18, 19). One transposon (Tn*1*) inserted 6 bp into the predicted coding region of BF638R_3923 (*ubb*) (Fig. 2B) and abrogated its ability to antagonize strain CL03T00C08. The second mutant (Tn*8*) had the transposon inserted 76 bp downstream from *ubb*, resulting in severely attenuated activity. We analyzed our previously generated high-throughput RNA sequencing (RNA-Seq) data on *in vitro*-grown mid-log-phase *B. fragilis* NCTC 9343, which contains a *ubb* genetic region identical to that of strain 638R (18, 19). These analyses predicted that the *ubb* transcript begins just following the −7 region of the *Bacteroides* sigma 70 binding site (20) and terminates 140 bp downstream from the *ubb* stop codon (Fig. 2B). Based on this prediction, Tn*8* has the transposon inserted within the *ubb* transcript. We tested whether these transposon mutants retained the ability to antagonize three other strains sensitive to 638RΔ1646 and found that growth inhibition of these strains was also abrogated or severely attenuated (Fig. 2A). The RNA-Seq data revealed that *ubb* is the 47th most highly expressed gene of the NCTC 9343 genome, with a fragments per kilobase of transcript per million mapped reads (FPKM) value of 7,453.

To confirm that the transposon insertions in the *ubb* transcript accounted for the loss of toxic activity, an internal deletion mutant with the deletion of *ubb* was created in the 638RΔ1646 background. This strain lost the ability to antagonize the four sensitive strains tested, and the toxic activity was restored when the gene was cloned into a *Bacteroides* expression vector and added to the deletion mutant in *trans* (Fig. 2C). Therefore, *ubb* is required for the antimicrobial activity against these strains.

### *B. fragilis* ubiquitin is the inhibitory factor.

**FIG 3:**
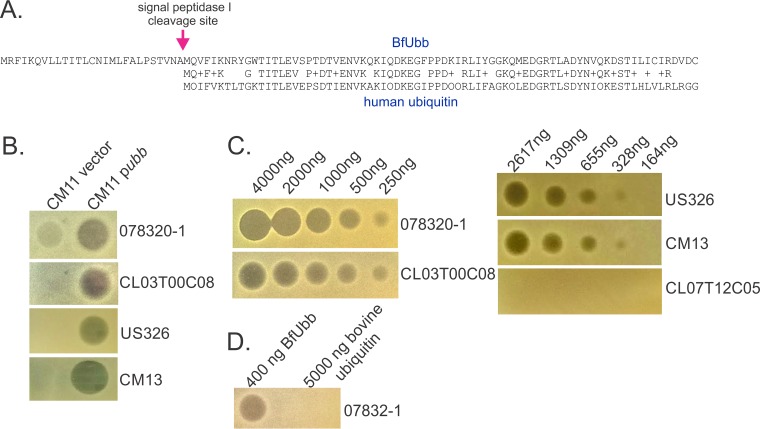
BfUbb inhibits growth. (A) Alignment of BfUbb with human ubiquitin, showing the extended N-terminal SpI signal sequence of BfUbb. (B) Agar spot assays displaying the sensitivity/resistance profiles of four *B. fragilis* strains exposed to *B. fragilis* CM11 with an empty vector or *ubb* in *trans*. (C) Agar spot assays showing inhibition activities of dilutions of synthesized 76-aa BfUbb against four sensitive strains and one resistant strain. (D) Agar spot assay showing sensitivity of *B. fragilis* strain 0878320-1 to BfUbb versus purified bovine ubiquitin.

BfUbb was previously shown to have a 27-amino-acid (aa) signal sequence and to be secreted extracellularly (18), even though the gene is annotated as a smaller open reading frame (ORF) without this sequence. Alignment of the mature 76-aa BfUbb with the 76-aa human ubiquitin shows that the similarity begins immediately after the signal sequence and the proteins are 84% similar along their lengths, with the exception of the last 4 amino acids (Fig. 3A). The fact that BfUbb is secreted suggests that it may mediate the toxic activity itself, either directly or indirectly. To eliminate the possibility that BfUbb may be modifying or acting on another gene or gene product of strain 638R that mediates the activity, we placed *ubb* in *trans* in *B. fragilis* strain CM11, which does not have BfUbb activity and is also not sensitive to it (Table S1). We found that the acquisition of *ubb* by strain CM11 conferred upon it the ability to antagonize BfUbb-sensitive strains (Fig. 3B). These data also suggest that BfUbb-producing strains do not require an immunity protein for protection, as CM11 was not noticeably affected by the addition of *ubb*.

To further establish that BfUbb is the inhibitory factor of strain 638R, we had the 76-aa BfUbb peptide (lacking the signal sequence) synthesized and tested it in the assay. We found that the synthesized protein has very potent activity against sensitive strains, where as little as 250 ng of the protein resulted in a detectable zone of inhibition (Fig. 3C). Strain *B. fragilis* CL07T12C05, which is not affected by wild-type 638R, is not inhibited by the BfUbb peptide (Fig. 3C). In addition, purified 76-aa bovine ubiquitin, which is identical to human ubiquitin, has no activity against these strains, even when a much greater dose is used (Fig. 3D). We also tested the remaining 36 *B. fragilis* strains used in this study for growth inhibition by the BfUbb peptide. Of the 40 strains analyzed, 13 are inhibited by BfUbb (Table S1). All of these strains are antagonized by the 638RΔ1646 mutant, as expected (Table S1). Unlike our findings with BSAP-1, where the *B. fragilis* strains analyzed either produce the toxin or are sensitive to it, there are many *B. fragilis* strains that do not synthesize BfUbb and are also not sensitive to it (Table S1).

### Species-wide analysis of the genetic architecture of the *ubb* region.

**FIG 4:**
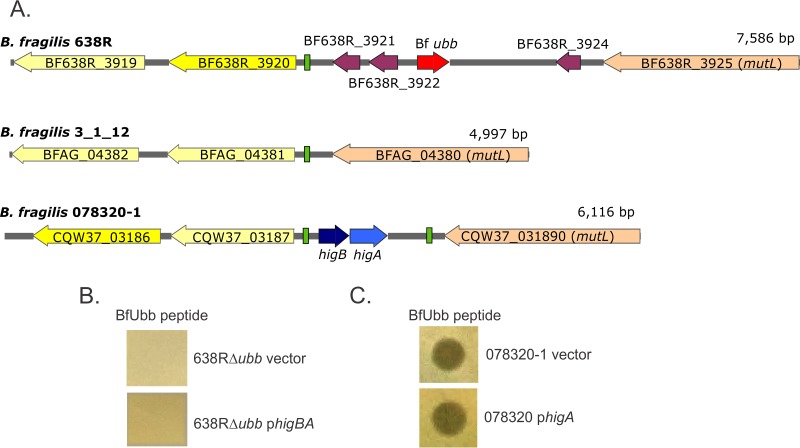
Heterogeneity of the *B. fragilis* genome in the *ubb* genetic region. (A) Gene maps of three representative *B. fragilis* strains showing the three predominant genetic types in the *ubb* or corresponding regions. *B. fragilis* strains each have a similar *mutL* (orange), and the DNA between each of the three genetic types begins to diverge 39 bp downstream from this gene. The genes colored yellow encode proteins with β-propeller motifs that are divergent even within a genetic type. The green boxes indicate a 52-bp element that is present in each of the three genetic types and is identical between strain 638R and 078320-1, with a few mismatches in strain 3_1_12. The *B. fragilis* 638R gene map (top) shows that *ubb* is contained in a genetic region likely acquired with three other small genes. The *B. fragilis* 3_1_12 gene map (middle) shows that a few *B. fragilis* genomes have no genes inserted in this region. The *B. fragilis* 078320-1 gene map (bottom) shows the most predominant *B. fragilis* genetic type, where *higBA* genes are present in the divergent region along with an additional copy of the 52-bp element downstream from *mutL*. (B) Agar spot assay of BfUbb peptide overlaid with *B. fragilis* CM11 containing an empty vector or the vector expressing *higBA*. (C) Agar spot assay of the BfUbb peptide overlaid with sensitive strain *B. fragilis* 078320-1 containing an empty vector or the vector overexpressing the antitoxin-encoding gene *higA*.

To investigate the diversity of the *ubb* genetic region among *B. fragilis* strains, we analyzed the *ubb* region or the corresponding region in strains lacking *ubb*. We analyzed 97 *B. fragilis* genomes contained in our curated genome database and retrieved and analyzed DNA from *mutL* to the gene encoding the first protein with a β-propeller motif (Fig. 4A). This analysis revealed that this area of the *B. fragilis* genome is heterogeneous, with three major genetic types identified (Fig. 4A). Each genome has a similar *mutL*; however, the DNA between strains begins to diverge significantly 39 bp downstream from this gene. Of the 97 genomes analyzed, 13 contain a *ubb* and surrounding DNA nearly identical to that of strain 638R (Fig. 4A, top). Seventy-three of the strains completely lack *ubb* and in its place have two genes encoding a toxin-antitoxin pair of the HigBA family (Fig. 4A, bottom). Eleven strains do not have any genes present in this region (Fig. 4A, middle). Each of the genomes encodes proteins with β-propeller motifs outside the divergent region (Fig. 4A, genes colored yellow), but these sequences are not conserved, even within a genetic type. A 52-bp direct-repeat element (Fig. 4A, green boxes) was identified flanking the *higBA* genes, one located 61 bp upstream from a β-propeller-encoding gene and the other 257 bp downstream from *mutL*. A single copy of this 52-bp element is present in the other two genetic types, in both cases 61 to 67 bp upstream from their respective β-propeller-encoding genes (Fig. 4A). The 52-bp element in *ubb*-containing genomes is an exact match with the element in the *higBA* genetic group. However, the element differs somewhat in the genomes with no genes in the region; for example, strain 3_1_12 has 8 mismatches. The other small genes unique to the *ubb* genetic region do not encode proteins that appear to be of eukaryotic origin: BF638R_3921 is a peptidase of the S41 superfamily, BF638R_3922 is the N terminus of a truncated β-propeller protein, and BF638R_3934 is a hypothetical protein with no predicted function. Comparisons of genomes within a genetic group revealed that the genomes without any genes between *mutL* and the downstream β-propeller-encoding gene (represented by strain 3_1_12 in Fig. 4A) are the most divergent within a group. To determine whether the *ubb*-containing genomes may be more phylogenetically related to each other than to the genomes of the other two genetic variants, we analyzed by BLAST all 97 genomes for their phylotype of five conserved genes that are commonly used for phylogenetic analyses: *dnaJ*, *groEL*, *gyrB*, *recA*, and *rpoB*. It was shown many years ago that the species *B. fragilis* is comprised of two genetic groups, with each group having distinct β-lactamase-encoding genes, one with *cepA* and the other containing *cfiA* (*ccrA*) (21–23). In these analyses, we also found that *B. fragilis* genomes segregate into two distinct branches and correlate perfectly with the presence of *cepA* or *cfiA*. In analyzing our three genetic types in the *ubb* region, we found that the *ubb*-containing genomes are indistinguishable from genomes containing the *higBA* toxin-antitoxin pair using this phylotyping method. Both genetic types contain *cepA*, and the five conserved genes are >99.6% identical to each other. However, the 11 *B. fragilis* genomes that do not have any genes in this region are all of the other subspecies, those which contain *cfiA*. The five conserved genes are only 90 to 92% identical to the orthologous gene in *B. fragilis* genomes containing *ubb* or *higBA*.

Both BSAP-1 and BSAP-2 target surface molecules in sensitive cells encoded by genes that were replaced by DNA containing the incoming BSAP-encoding gene. Therefore, the BSAP receptor genes are in the same genetic region of sensitive strains as the BSAP gene in producing strains. We therefore considered that BfUbb may be affecting the HigBA toxin-antitoxin (TA) system, which is not present in Bf*ubb*-containing strains. Among possible mechanisms for such an activity is the binding of BfUbb to the HigA antitoxin so that it can no longer interact with the HigB toxin to prevent self-intoxication. Three lines of evidence suggest that the toxicity of BfUbb may not involve the HigBA TA system. We first attempted to delete either *higBA* or just the *higB* toxin gene from several sensitive *B. fragilis* strains. Despite repeated attempts, we were unable to construct these deletions, possibly due to the difficulties of creating deletion mutants in some *B. fragilis* strains or an intolerance to the deletion of these genes. In lieu of a deletion mutant, we cloned *higBA* into a *Bacteroides* expression vector with a constitutive promoter, thereby eliminating the normal regulation of these genes, and this construct was conjugated into strain 638RΔ*ubb*. The presence of constitutively expressed *higBA* did not render 638RΔ*ubb* sensitive to BfUbb (Fig. 4B). We also cloned *higA* into a vector for high gene expression in *Bacteroides* to determine whether excess quantities of the antitoxin could overcome the effects of BfUbb in a sensitive strain. We observed no difference in the ability of various quantities of BfUbb to antagonize *B. fragilis* 078320-1 in the agar spot assay when this sensitive strain contained either the empty vector or the highly expressed antitoxin gene (Fig. 4C, showing results for one concentration). Another finding indicating that the HigBA system may not be the target of BfUbb is that we identified four *B. fragilis* strains with *higAB* genetic regions nearly identical to those of other sensitive strains, and yet, these four strains are not sensitive to BfUbb (Table S1).

### Analysis of human gut metagenomic data sets for *ubb.*

Among the 97 sequenced *B. fragilis* genomes in our curated genome collection, 13 genomes contain *ubb*. These analyses estimated the frequency of *ubb* in *B. fragilis* genomes at approximately 13%. To determine the frequency of *ubb* in the human gut microbiota, we analyzed the 3CGC human gut metagenomic set, a subset of the recently compiled integrated gene catalog (IGC) that contains 1,267 metagenomes, for the presence of *ubb*. We detected evidence of the species *B. fragilis* in 370 of these metagenomes, of which *ubb* was detected in 44 (Table S2). *ubb* was only detected in metagenomes identified as containing *B. fragilis* (Table S2). Based on these data, approximately 12% of metagenomes with evidence of *B. fragilis* contain *ubb*, a percentage relatively consistent with that of our sequenced genome collection. In addition, we found numerous metagenomes that contain both the BSAP-1 encoding gene and *ubb* and some metagenomes that contain one or the other. In total, 130 metagenomes contain at least one of the two secreted antimicrobial-protein-encoding genes, and 20 contain genes encoding both BSAP-1 and BfUbb.

10.1128/mBio.01902-17.2 TABLE S2  Identification of *B. fragilis* and *ubb*, *higA*, *higB*, and BF638R_1646 (*bsap1*) in human gut metagenomes comprising subset 3CGC of the integrated gene catalog (IGC). Download TABLE S2, XLSX file, 0.1 MB. Copyright © 2017 Chatzidaki-Livanis et al. 2017 Chatzidaki-Livanis et al. This content is distributed under the terms of the Creative Commons Attribution 4.0 International license.

## DISCUSSION

Ubiquitin is found in eukaryotic organisms from fungi to humans. Ubiquitin is attached to eukaryotic proteins by a series of three enzymes that create isopeptide bonds. Ubiquitination of these substrate proteins regulates their cellular fate in numerous and distinct ways. Ubiquitination or polyubiquitination of eukaryotic proteins can affect protein degradation (reviewed in reference 24), localization (25), activity, and interactions with other molecules (26). Ubiquitination of proteins has not been demonstrated in bacteria, largely due to the fact that most bacteria do not produce a eukaryotic-like ubiquitin. However, some pathogenic bacteria produce proteins that alter eukaryotic cell function by interfering with the ubiquitin signaling pathways in host cells (reviewed in reference 27). Therefore, coevolution of bacteria with eukaryotic hosts has resulted in ubiquitin-related processes involved in toxicity, where either the ubiquitin molecule itself is involved in antagonistic interactions or, as described above, bacterial effectors alter host ubiquitin processes.

*B. fragilis* is one of a few bacteria that encode a eukaryotic-like ubiquitin. BfUbb is an interesting ubiquitin ortholog with many similarities to human ubiquitin but with some important distinctions. One of the major differences is in the C termini of these molecules. The last 4 amino acids are distinct, and BfUbb lacks the critical terminal glycine residue involved in the isopeptide linkage to substrate proteins in the eukaryotic system. Therefore, BfUbb would not serve as a substrate for eukaryotic E3 ubiquitin ligase. Another distinction is that BfUbb is glycosylated; Patrick et al. (18) previously noted that BfUbb has two *Bacteroidetes* glycosylation sites (28). Our prior study showed that all analyzed secreted proteins of *B. fragilis* that have glycosylation motifs are in fact glycosylated (29), strongly suggesting that BfUbb is glycosylated. As synthetic BfUbb has potent activity, glycosylation of this protein is not necessary for its ability to antagonize target strains. We previously showed that glycosylation of some *Bacteroides* secreted proteins increases their stability (28); therefore, glycosylation of BfUbb may be important in the context of the bacterial cell.

One of the most interesting distinctions of BfUbb is the N-terminal signal sequence dictating its processing by signal peptidase I and subsequent secretion. The cleavage site of this signal peptide is located such that mature BfUbb aligns exactly with the first amino acid of its human counterpart (Fig. 2B). Although mammalian ubiquitin does not contain such a signal sequence, there are ubiquitin orthologs described in several nematode species with this feature. The best described is produced by the nematode *Globodera rostochiensis*, a parasite of plants that creates a syncytium for successful parasitism. The secreted ubiquitin of this organism is produced exclusively in the gland of the nematode and serves as an effector for host syncytium formation (30). In addition to the N-terminal signal sequence, the nematode ubiquitin has a 12-aa C-terminal extension that is cleaved from the ubiquitin molecule in the plant. The 12-aa peptide suppresses effector-triggered immunity. The remaining core ubiquitin molecule also plays a role in parasitism, possibly by perturbing ubiquitin levels, thereby affecting the host 26S proteasome (30). Therefore, the unique ubiquitin molecules produced by some nematodes are similar to BfUbb in that they are secreted and result in effector/toxic activity in recipient cells.

The source from which *ubb* was acquired is not clear from existing genomic sequences. There are several amoeba-infecting giant viruses that encode ubiquitin molecules (31, 32) that are the closest orthologs of mature BfUbb in the databases. One such ubiquitin-encoding giant virus was identified in human stool (33). Giant virus-infected cells are on occasion coinfected with small virophage that are eukaryotic viruses but have properties of prokaryotic phage and are predicted to transfer genes between giant viruses during coinfection (34). It is possible that such a virus or phage may have introduced an ortholog of *ubb* into the *B. fragilis* genome. We did not detect any obvious signs in the *B. fragilis* genomes to hint at how this region was acquired. However, we did identify a 52-bp element in the divergent regions of all three genetic variants (Fig. 4A). This 52-bp element is duplicated in *higBA*-containing genomes and flanks these genes. Therefore, this region may represent an integration site for chromosomal insertions.

How BfUbb antagonizes specific *B. fragilis* strains is not readily obvious from the protein sequence or genomic analyses. A potential target is the antitoxin protein of the HigBA toxin-antitoxin system that is present in the majority of non-*ubb*-containing *B. fragilis* strains. Our experimental and genomic data did not confirm a role for the HigBA TA system in the activity of BfUbb, but its involvement has also not been excluded. BSAP-1 and BSAP-2 each contain an MACPF domain and bind surface receptors, leading to pore formation. BfUbb may function by a completely different mechanism. For BSAP-1, *B. fragilis* strains typically produce the toxin or are sensitive to it. This occurs because BSAP-1 binds a surface protein necessary for gut colonization, and in BSAP-1-producing strains, the gene encoding the protein conferring sensitivity is replaced by an ortholog that serves its function in gut colonization but also renders the strain resistant to the BSAP-1. Therefore, most non-BSAP-1-producing strains have the BSAP-1 surface target by default. For BfUbb, no such pattern emerged, as we found many strains without *ubb* that were not sensitive to it. Our data also suggest that, similar to BSAP-1 and BSAP-2, BfUbb does not require an immunity protein to protect the producing cell. Therefore, it is likely that sensitive cells contain a specific molecule that BfUbb targets rather than certain *B. fragilis* strains producing an immunity protein for resistance. It is possible that BfUbb is transported into cells by a protein-specific nutrient uptake system, where it would act on an intracellular target rather than at the bacterial surface. Continued analysis of BfUbb will likely reveal a novel mechanism of action.

This is the first bacterially produced eukaryotic-like ubiquitin molecule shown to intoxicate bacterial cells, whether directly or indirectly. To date, the three described secreted antimicrobial proteins produced by *Bacteroides* all have eukaryotic-like features. We previously showed that *Bacteroides* species synthesize another mammalian-like enzyme, termed Fkp, that charges fucose with GDP for its addition to surface glycans of these bacteria. Fkp is necessary for the bacteria to colonize the mammalian gut (35). These secreted antimicrobial molecules are additional examples of these host-associated bacteria likely acquiring and adapting eukaryotic molecules to increase their fitness in the human gut.

The importance of antagonism to bacterial colonization of the mammalian gut is evident by the fact that a single *B. fragilis* strain produces not only a T6SS that targets nearly all *Bacteroidales* species (13) but also at least two secreted antimicrobial proteins to further antagonize strains of the same species. Identifying and characterizing molecules of gut bacteria that mediate competitive interactions will allow the rational design of engineered probiotic-type bacteria to successfully colonize the host to deliver health-promoting functions.

## MATERIALS AND METHODS

### Primers.

All primers used in this study are listed in Table S3 in the supplemental material.

10.1128/mBio.01902-17.3 TABLE S3  Primers used in this study. Download TABLE S3, DOCX file, 0.01 MB. Copyright © 2017 Chatzidaki-Livanis et al. 2017 Chatzidaki-Livanis et al. This content is distributed under the terms of the Creative Commons Attribution 4.0 International license.

### Bacterial strains and growth conditions.

The *Bacteroides* strains used in this study were previously described (1, 36). All *Bacteroides* strains were grown in supplemented basal medium (37) or on supplemented brain heart infusion (BHIS) plates. Antibiotics (5 µg/ml erythromycin or 3 µg/ml tetracycline) were added where indicated below. *Escherichia coli* strains were grown in L broth or L plates with antibiotics added where appropriate (100 µg/ml ampicillin, 100 µg/ml trimethoprim, and 50 µg/ml kanamycin).

### Agar spot test for growth inhibition analysis.

The ability of *Bacteroides* strains to inhibit the growth of other strains was assayed using the agar spot test (38). In brief, *Bacteroides* strains were resuspended from plates into phosphate-buffered saline (PBS) at a density of approximately 10^10^/ml, and 5-µl volumes were spotted onto BHIS plates and grown anaerobically at 37°C overnight. The bacteria were removed with swabs, and the residual bacteria remaining on the plates were killed by exposing them to chloroform vapor for 15 min. Strains to be tested for growth inhibition were grown to an optical density at 600 nm (OD_600_) of 0.6, and then 100-µl amounts were mixed with 4 ml top agar and overlaid onto the chloroform-treated plates. The zones of inhibition were analyzed after anaerobic overnight incubation at 37°C.

### Growth inhibition assays using synthetic BfUbb, mammalian ubiquitin, and His-tagged BSAP-1.

The 76-aa BfUbb peptide corresponding to the mature molecule without the signal sequence was synthesized by LifeTein (Hillsborough, NJ). Agar overlay assays using synthetic BfUbb were performed the same as the regular agar spot assays except that the purified protein in PBS was added to the BHIS plates (2.6 µg in a 5-µl volume unless otherwise indicated), allowed to dry, and then overlaid with strains as described above. Purified bovine ubiquitin (76 aa, >98% pure) from erythrocytes was purchased from Sigma (U6253) and resuspended in PBS. Five micrograms of protein in 5 µl was spotted onto the BHIS plates for the overlay assay. The N-terminally His-tagged BSAP-1 protein was purified and processed as previously described (16). For the agar overlay assay, 2.5 µg of His-BSAP in a 5-µl volume of PBS was added to the plates and allowed to dry before performing the overlays.

### Transposon mutagenesis and deletion of BF638R_3923 (*ubb*).

Random mutagenesis of *B. fragilis* 638RΔ1646 was performed using the transposon-containing plasmid pYT646b as described previously (39), using tetracycline selection. The insertion sites of transposon mutants were identified by cloning the junctional DNA as described previously (16, 39).

A deletion mutant with the deletion of *ubb* was constructed such that 255 bp of the 312-bp gene was removed. DNA segments upstream and downstream from the region to be deleted were PCR amplified, and the PCR products were digested with BamHI and EcoRI and cloned by three-way ligation into the BamHI site of pNJR6 (40). The resulting plasmid was conjugally transferred into wild-type *B. fragilis* 638R or 638RΔ1646 using helper plasmid R751, and cointegrates were selected by erythromycin resistance. Following growth under nonselective conditions, erythromycin-sensitive colonies were screened by PCR for the mutant genotype.

### Cloning *ubb* into a conjugal expression vector.

*ubb* was PCR amplified using primers with BamHI ends (Table S3). The PCR product was digested with BamHI, cloned into the BamHI site of *Bacteroides* expression vector pFD340 (41), and screened for correct orientation in relation to the plasmid-borne promoter. The resulting plasmid was conjugated into *B. fragilis* 638RΔ1646 and *B. fragilis* CM11 by conjugal mating using an *E. coli* strain containing helper plasmid RK231 and selected by acquisition of erythromycin resistance.

### Cloning of the *higBA* toxin-antitoxin genes.

The putative toxin-antitoxin genes HMPREF1067_00095 and -96 were PCR amplified from sensitive strain CL03T12C07, cloned into the BamHI site of pFD340, and screened for correct orientation in relation to the plasmid-borne promoter. The antitoxin gene (HMPREF1067_00096) was cloned into pMCL140 (42), a *Bacteroides* expression vector for high expression of cloned genes. Both plasmids were conjugated into *Bacteroides* strains as described above.

### Determination of *ubb* transcript size and expression level.

We reanalyzed the two biological replicates of wild-type *B. fragilis* NCTC 9343 from our previously generated RNA-Seq data (13) by first adapter and quality trimming the two sets of paired-end reads using BBDuk (see “Genome sequencing of additional *B. fragilis* strains” below). Read alignment, transcript prediction, and statistical calculations, including normalization and measures of relative expression (fragments per kilobase of transcript per million mapped reads [FPKM]), were achieved using HISAT2 (version 2.1.0) (43), samtools (version 1.6) (44), and StringTie (version 1.1.3) (45). We used the sequence information for *B. fragilis* NCTC 9343 available from NCBI (GenBank accession number NC_003228) as a scaffold, except that we corrected the start coordinate of the *ubb* gene (BF9343_3779) to reflect the true beginning of the open reading frame.

### Detection of *B. fragilis* and *B. fragilis* genes in human gut metagenomes.

Metagenomic analyses were performed using a subset (3CGC) of the recently compiled integrated gene catalog (IGC) (46). This subset comprises 1,267 human gut metagenomes. To detect *B. fragilis* in these metagenomes, we used DNA sequences of single-copy genes known previously to differentiate *B. fragilis* from other species, namely, *dnaJ*, *groL*, *gyrB*, *recA*, and *rpoB*. The DNA sequences of these five genes were collected from each of the three *B. fragilis* strains with unique genetic types in the *ubb* or corresponding region. The genes from *B. fragilis* 638R are BF638R_1741 (*dnaJ*), BF638R_3250 (*groL*), BF638R_0298 (*gyrB*), BF638R_1245 (*recA*), and BF638R_4052 (*rpoB*); the genes from *B. fragilis* 078320-1 are CQW37_03448 (*dnaJ*), CQW37_03630 (*groL*), CQW37_02683 (*gyrB*), CQW37_01563 (*recA*), and CQW37_01330 (*rpoB*); and the genes from *B. fragilis* 3_1_12 are BFAG_01078 (*dnaJ*), BFAG_03510 (*groL*), BFAG_02716 (*gyrB*), BFAG_00519 (*recA*), and BFAG_04295 (*rpoB*). These fifteen gene sequences were used as queries against a blastn database created using the makeblastdb program. The output from the blastn command (executed with switches -task megablast -evalue 1e−5 -dust no -best_hit_score_edge 0.05 -best_hit_overhang 0.25) was parsed, and the best hit (by highest bit score) returned from each metagenome was retained if that metagenome had a hit reaching the threshold levels indicated. Hits that survived the filter cutoff values were defined as evidence of the presence of *B. fragilis* in the subject metagenome (Table S2). The search for *ubb*, *higBA*, and the BSAP-1-encoding gene in the metagenomes was performed in the same way, using the DNA sequences of CQW37_03188 and CQW37_03189 (the *higB* and *higA* genes, respectively, from *B. fragilis* 078320-1), BF638R_3923 (the *ubb* gene from *B. fragilis* 638R, including the region encoding the heretofore unannotated signal sequence), and BF638R_1646 (the BSAP-1-encoding gene from *B. fragilis* 638R) as queries (Table S2).

### Genome sequencing of additional *B. fragilis* strains.

Chromosomal DNA from *B. fragilis* strains 12905, CL04T03C20, US326, CM13, 1284, and 078320-1 was fragmented using the Covaris S2 instrument and analyzed for fragment distribution with a high-sensitivity D1K TapeStation machine and for sufficient quantity by a SYBR quantitative PCR (qPCR) assay. The DNA was sequenced using an Illumina MiSeq sequencer, producing paired-end reads of 150 bp. Genomic sequencing was performed by the Biopolymers Facility, Harvard Medical School. The raw paired Illumina reads were processed to remove adapter sequences and quality trimmed using BBDuk, part of the BBTools (version 37.50) suite of programs distributed by the Department of Energy’s Joint Genome Institute (https://jgi.doe.gov/data-and-tools/bbtools/). NCBI’s UniVec_Core database (build 10.0) was downloaded (ftp://ftp.ncbi.nlm.nih.gov/pub/UniVec), entries originating in GenBank were removed, and the Illumina reads were further screened against this data set using blastn, removing any read that returned a significant hit. The reads passing these screens (including orphans) were used to assemble the genomes. Velvet Optimizer (version 2.2.5, http://www.vicbioinformatics.com/software.velvetoptimiser.shtml) was utilized to determine the optimal *k* value (among other settings), and the genomes were assembled *de novo* using Velvet 1.2.10 (47). The draft genomes were annotated using an in-house-customized version of Prokka version 1.12 (48).

### Accession numbers.

Genomes were deposited in GenBank under BioProject identification number (ID) PRJNA413027 and the following BioSample IDs: *B. fragilis* 1284, SAMN07735158; *B. fragilis* 12905, SAMN07735159; *B. fragilis* 078320-1, SAMN07735199; *B. fragilis* CL04T03C20, SAMN07735200; *B. fragilis* CM13, SAMN07735201; and *B. fragilis* US326, SAMN07735202.
