# Supplementary material for: Gut Symbiont Bacteroides fragilis Secretes a Eukaryotic-Like Ubiquitin Protein That Mediates Intraspecies Antagonism
Source: mBio. 2017 Nov 28;8(6):e01902-17. doi: 10.1128/mBio.01902-17 (PMC5705921; doi:10.1128/mBio.01902-17)
Supplement: TABLE S1 [file mbo006173610st1.docx]

| **Table S1.**  Ability of *B. fragilis* 638R, 638RΔ1646 mutant, and toxins to inhibit the growth of *B. fragilis* strains | | | | | | | |
| --- | --- | --- | --- | --- | --- | --- | --- |
|  | **growth inhibition by** | | | |  |  |  |
| **overlay strain** | **638R** | **Δ1646 (Δ*bsap-1*)** | **His-BSAP-1** | **BfUbb peptide** |  | **genome sequence** | ***higBA* or *ubb*** |
| CL03T00C08 |  |  |  |  |  | ***** | *higBA* |
| CL05T12C13 |  |  |  |  |  | ***** | *higBA* |
| CM13 |  |  | faint |  |  | this study | *higBA* |
| 20793-3 |  |  |  |  |  | ***** | *higBA* |
| 078320-1 |  |  |  |  |  | this study | *higBA* |
| US326 |  |  |  |  |  | this study | *higBA* |
| S36-L11 |  |  |  |  |  | ***** | *higBA* |
| J-38-1 |  |  |  |  |  | ***** | *higBA* |
| 2_1_16 | faint | faint |  |  |  | ***** | *higBA* |
| 12905 |  |  |  |  |  | this study | *higBA* |
| CL07T12C05 |  |  |  |  |  | ***** | *higBA* |
| 9343 |  |  |  |  |  | ***** | *ubb* |
| I1345 |  |  |  |  |  | ***** | *ubb* |
| CL04T03C20 |  |  |  |  |  | this study | *ubb* |
| 1284 |  |  |  |  |  | this study | *ubb* |
| Korea 419 |  |  |  |  |  | ***** | *ubb* |
| 3_1_12 |  |  |  |  |  | ***** | no |
| 1279153I |  |  |  | faint |  |  |  |
| 419 |  |  |  |  |  |  |  |
| LM001 |  |  |  |  |  |  |  |
| 13141 |  |  |  |  |  |  |  |
| B117 |  |  |  |  |  |  |  |
| 26877 |  |  | faint |  |  |  |  |
| LM16 |  |  |  |  |  |  |  |
| 12877810I |  |  |  |  |  |  |  |
| 1281550I |  |  |  |  |  |  |  |
| CM11 |  | faint |  |  |  |  |  |
| LM46 | faint | faint |  |  |  |  |  |
| 26783 |  |  |  |  |  |  |  |
| 38310 |  | faint |  |  |  |  |  |
| US357 |  | faint |  |  |  |  |  |
| LM41 | faint |  |  |  |  |  |  |
| 1277476 | faint | faint | faint |  |  |  |  |
| DSM2151 | faint |  |  |  |  |  |  |
| US2244 |  | faint |  |  |  |  |  |
| 379 |  |  |  |  |  |  |  |
| B124 |  |  |  |  |  |  |  |
| LM2 |  |  |  |  |  |  |  |
| LM8 |  |  |  |  |  |  |  |
| LM36 |  |  |  |  |  |  |  |
| blue shaded box indicates the overlay strain was growth inhibited by the strain/protein | | | | | | |  |
| designation of faint indicates a hazy zone of inhibition | | | |  |  |  |  |
| The presence of *higBA* or *ubb* in sequenced genomes is shown in the right column | | | | | | |  |
