# Supplementary material for: Gut Symbiont Bacteroides fragilis Secretes a Eukaryotic-Like Ubiquitin Protein That Mediates Intraspecies Antagonism
Source: mBio. 2017 Nov 28;8(6):e01902-17. doi: 10.1128/mBio.01902-17 (PMC5705921; doi:10.1128/mBio.01902-17)
Supplement: TABLE S3 [file mbo006173610st3.docx]

| **Table S3.** Primers used in this study | | |  |
| --- | --- | --- | --- |
|  | |  |  |
| Purpose | Primer | | Primer sequence^a^ |
|  |  | |  |
| Deletion of BF638R_3923 | left flank forward | | tcagggatccgtcattatcattctcgtgcagact |
|  | left flank reverse | | gtttgaattcgtcaaaagtacttgtttaataaatctcatttg |
|  | right flank forward | | GTCAGAATTCAATTTGCATAAGAGATGTCGACTG |
|  | right flank reverse | | CAGTGGATCCCACATATCCGTGTGCTCTTTGAC |
|  |  | |  |
| Expression of BF638R_3923 | forward | | TCCCGGATCCCAGATAACTTATATTTGTCCC |
|  | reverse | | TGGCGGATCCTTTTCTATTTATGCGTAATTTAGAACGGT |
|  |  | |  |
| Expression of *higBA*  (toxin-antitoxin genes) | forward | | AAAGGGATCCGAAAAAGTGTCTTGCCTTCATATGAAA |
|  | reverse | | CTAAGGATCCGGCGTGAAACCTAAAAATGGAATC |
|  |  | |  |
| Overexpression of *higA*  (antitoxin gene) | forward | | CGTAGGATCCTGATAACAAAGATTGTTCAAATGC |
|  | reverse | | AACAGGATCCCACGCCTAAAATATATCACGACAA |
|  |  | |  |

^a^ Restriction sites are underlined.
